# Toxic Effect of Destruxin A on Abnormal Wing Disc-Like (SLAWD) in *Spodoptera litura* Fabricius (Lepidoptera: Noctuidae)

**DOI:** 10.1371/journal.pone.0057213

**Published:** 2013-02-28

**Authors:** Xiang Meng, Junjie Hu, Xiaoxia Xu, Zeqing Wang, Qiongbu Hu, Fengliang Jin, Shunxiang Ren

**Affiliations:** 1 Engineering Research Center of Biological Control, Ministry of Education, South China Agricultural University (SCAU), Guangzhou, China; 2 Guangdong Entomological Institute, Guangzhou, China; 3 College of Life Science, Guangzhou University, Guangzhou, China; 4 Guangdong New Scene Biological Engineering Co.,LTD, Guangdong, China; University of Kentucky, United States of America

## Abstract

**Background:**

Destruxin A (DA) is a microbial insecticide with potent bioactivity against *Spodoptera litura* larvae. A previous proteomic analysis of *S. litura* (SL-1) cells exposed to DA showed the abnormal expression of wing disc-like protein of *S. litura* (SLAWD). To further understand the effect of DA on *SLAWD* expression, a functional study was carried out.

**Principal Findings:**

The SLAWD gene (*SLAWD*) was cloned and an open reading frame of 537 bp encoding a polypeptide of 178 amino acids was detected. Real-time fluorescence quantitative PCR (qRT-PCR) suggested that *SLAWD* is expressed in all developmental stages of *S. litura*, but expression was highest during the pupal and adult stages. RNAi knockdown of *SLAWD* expression in 6th-stage larvae was achieved by the microinjection of a specific double-stranded RNA (dsRNA). The results showed a significant decrease in *SLAWD* mRNA expression levels between the prepupal and adult stages. Interestingly, *SLAWD* expression was similarly down-regulated by treating 6th-stage larvae with DA. Growth- and development-related statistics confirmed the observed abnormalities in *S. litura* development after either RNAi or DA treatment.

**Conclusions:**

*SLAWD* appears to have a biosynthetic function in the pupal and adult stages of *S. litura*. The toxic effect of DA on *S. litura* development is due the negative effect of DA on *SLAWD* gene expression.

## Introduction

The imaginal discs of *Drosophila melanogaste*r are larval structures that form the epidermis of the adult head, thorax, and genitalia [1]. Normal imaginal disc development requires the coordinated activities of many genes. Mutations in these genes cause one or more pairs of discs to develop abnormally and are in many cases lethal [2], [3], [4], [5], [6]. In numerous holometabolous insect species, the wing imaginal disc gives rise to the adult wing. As one of the larval primordia, it develops slowly during the larval period but more rapidly after the wandering stage [7]. In a previous study, an abnormal wing disc gene (*AWD*) was identified in a genetic screen for genes involved in imaginal disc development [1], [8]. *AWD* of *Drosophila* is homologous to the *nm23* gene of mammals, whose expression is altered in metastatic tumors [9], [10], [11], [12]. Both genes encode a protein subunit with nucleoside diphosphate kinase (NDP kinase) activity [13], [14]. In a lepidopteran model in the silkworm *Bombyx mori*, *AWD* (*BmAWD*) expression is closely linked to wing development, as it controls the entire process of wing integrity and function [15]. Moreover, in this model, development of the wing imaginal disc is dependent on hormones and other factors present in the hemolymph [16], [17].

Worldwide, *Spodoptera litura* Fabricius (Lepidoptera: Noctuidae) is one of the most destructive phytophagous pests known; it infests cotton, vegetables, oilseed, and fiber crops [18], [19], [20]. Outbreaks of *S. litura* cause considerable agricultural and economic losses. However, with the widespread use of chemical pesticides, pest resistance has become an increasingly serious problem. Moreover, the improper use of several pesticides has raised awareness of their eco-environmental and human impacts. In the search for environmentally safe and sustainable alternatives, microbial insecticides represent ideal alternatives for integrated pest management programs [21].

Destruxins are microbial insecticides produced by different species of entomopathogenic fungi. These potent toxins exhibit bioactivity against *S. litura* larvae, *Bemisia tabaci* nymphs, and *Pieris brassicae* larvae [22], [23], [24]. Many factors determine the mechanisms of action of destruxins. Destruxins inhibit the hydrolytic activity of the V-type ATPase in the brush border membrane of the *G. mellonella* midgut [25], suppress the insect immune response [26], [27], and induce oxidative stress in *S. litura* by increasing the levels of superoxide radicals, including the NADPH-dependent form [24], [28].

In previous studies in our laboratory, a direct relationship was established between the production and insecticidal activity of destruxins [23], [29], [30]. In addition, we used high-resolution two-dimensional gel electrophoresis to analyze the toxicity of destruxin A (DA) and its target proteins in *S. litura* SL-1 cells [31]. However, it is currently unclear whether DA has specific effects on *SLAWD* expression. Thus, to better understand the effects of DA on *SLAWD* expression, we first cloned *SLAWD* and then used qRT-PCR to characterize its expression during development. Furthermore, we performed RNA interference (RNAi) knockdown of *SLAWD* expression by microinjecting a double-stranded RNA (dsRNA) into the larval hemocoel. Finally, by studying *SLAWD* expression in the presence of DA, we gained insights into the toxic effect of this microbial insecticide on *S. litura* development. The results of this study should help in developing new genetic approaches to insect pest control.

## Results

### Identification of the Gene for AWD in the *S. litura* Genome

**Figure 1:**
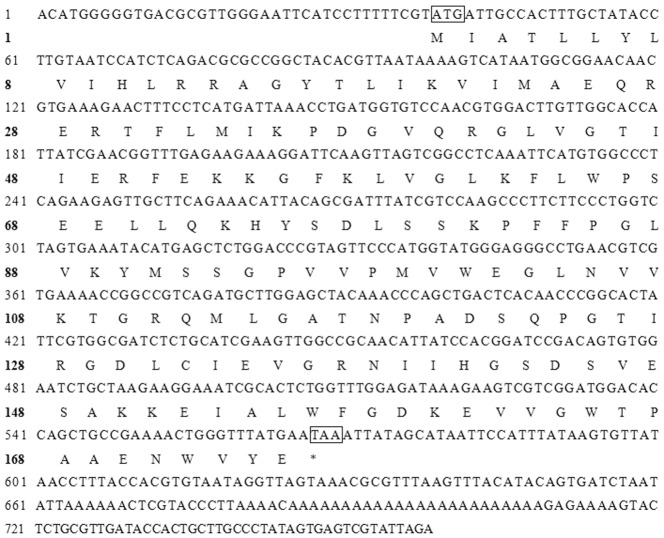
SLAWD nucleotide and amino acid sequences. Boxes indicate the initiator codon and terminator codon.

The full-length SLAWD cDNA sequence was obtained and deposited in GenBank under the accession no. JF690666. In total, 764 bp of the *SLAWD* transcript was sequenced, We detected an open reading frame (ORF) of 537 nucleotides with the potential to encode for a protein of 178 amino acids, with a predicted molecular mass of approximately 20.10 kDa and a pI of 8.94 (Fig. 1).

### Phylogenetic Analysis of AWD Genes and Proteins

**Figure 2:**
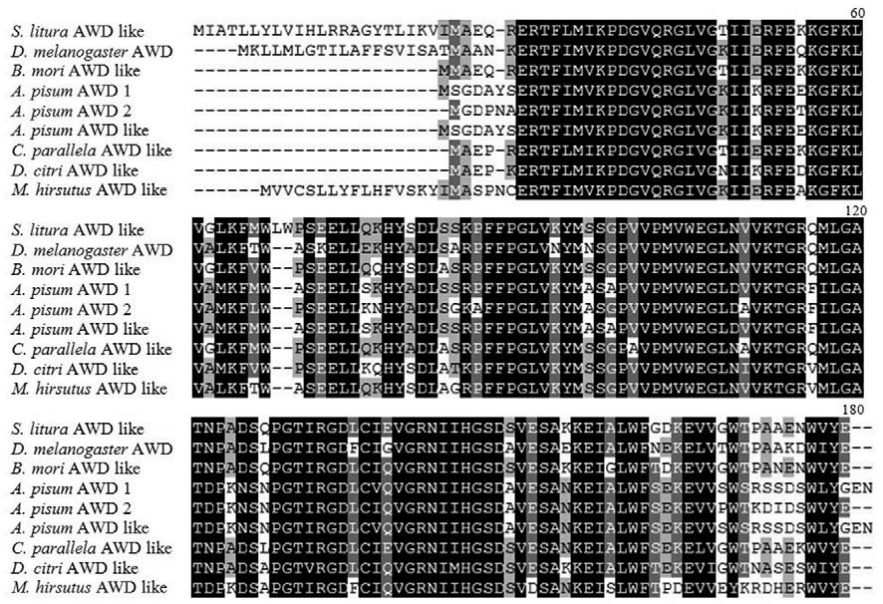
Multiple alignment of SLAWD and the translated amino acid sequences of AWD genes from other insects using ClustalW software.

**Figure 3:**
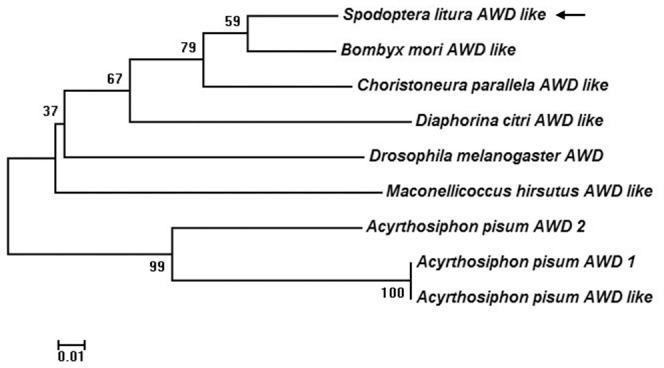
Phylogenetic analysis of insect AWD and AWD homologues. MEGA 4.0 was used to construct the phylogenetic tree. Bootstrap analyses from 1000 replications are shown by each branch. Arrows indicate SLAWD protein. Genbank accession numbers of the sequences are as follows: *D. melanogaster* AWD NP_476761.2; *B. mori* AWD like, GenBank: ABF51506.1; *A. pisum* AWD 1, NP_001119625.1; *A. pisum* AWD 2, NP_001119656.1; *A. pisum* AWD like, GenBank: ABD91521.1; *C. parallela* AWD like, GenBank: AAM53644.1; *D. citri* AWD like, GenBank: ABG81980.1; *M. hirsutus* AWD like, GenBank: ABM55663.1.

Alignment of the deduced amino acid sequence of SLAWD with sequences of AWD subunits from other insects showed that the predicted protein shares high homology with other known AWD subunits (Fig. 2). A phylogenic tree constructed using the full-length sequences of insect AWDs (Fig. 3) demonstrated high overall identity between the amino acid sequence of SLAWD and those of *Choristoneura parallela* abnormal wing disc-like (83%), *B. mori* abnormal wing disc-like (80%), and *D. melanogaster* abnormal wing disc-like (73%) proteins.

### Developmental Expression Pattern of *SLAWD*

**Figure 4:**
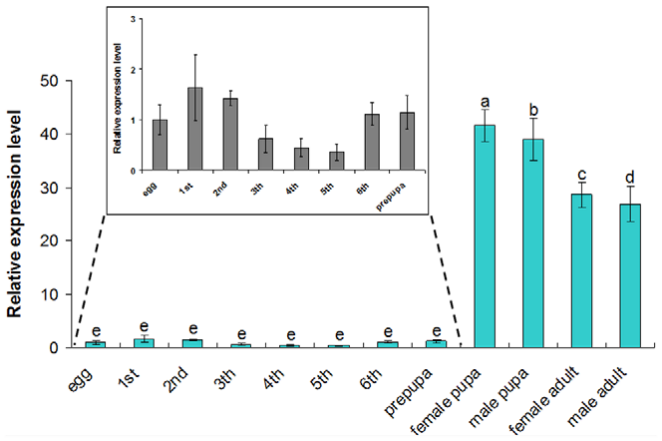
SLAWD expression during the different developmental stages of *S. litura*. The different letters above the columns indicate significant differences in *AWD* gene expression during *S. litura* development (*P*<0.05).

To determine whether the expression profiles of *SLAWD* changed during *S. litura* development, RNA was prepared from embryos, larvae (1^st^ to 6^th^ stages), prepupae, pupae, and adults (male and female), and a transcriptional analysis was carried out by qRT-PCR using gene-specific primer-pairs. AWD transcripts were detected at all developmental stages (Fig. 4), indicating the consistent expression of *SLAWD* throughout *S. litura* development; however, expression was higher in the pupal and adult stages and lower in the larval and prepupal stages. These results suggested a synthetic role for AWD in the pupal stage of *S. litura*.

### Efficiency of RNAi and DA Inhibition of *SLAWD*

RNAi experiments were carried out using dsRNAs that targeted different regions of the *SLAWD* gene to determine the role of *SLAWD* in *S. litura* growth and development. In addition, development was monitored in the presence of DA to test its effect on *SLAWD* expression. Normal development was disrupted following the microinjection of dsRNA or DA into 6^th^ instar larvae of *S. litura.*

#### Expression of SLAWD after RNAi or DA treatment

**Figure 5:**
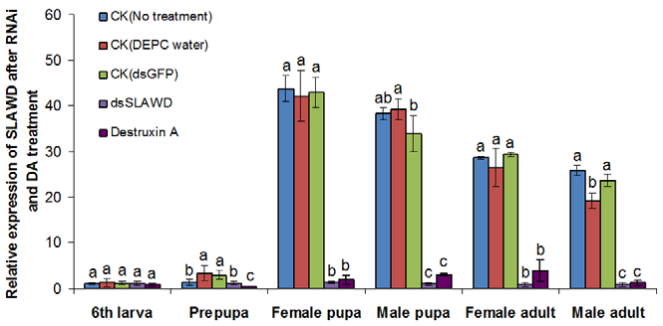
Analysis of the relative expression level of SLAWD during the different developmental stages of *S. litura* by qRT-PCR after RNAi or DA treatment. The different letters above the columns indicate significant differences in *SLAWD* expression after RNAi orDA treatment (*P*<0.05).

To determine the effects of dsRNA or DA on SLAWD expression during *S. litura* development, total RNA was isolated at different developmental stages of the insect after the microinjection of dsRNA or DA. A qRT-PCR analysis (Fig. 5) showed that *SLAWD* gene expression did not differ between the three controls (no treatment, DEPC water, dsGFP) and corresponded to that seen at different developmental stages of untreated *S. litura* larvae. After dsRNA or DA treatment, the relative expression of *SLAWD* was decreased to that of the prepupal phase, and decreased further between the pupal stage and the adult stage, but there was no obvious difference between the expression in 6^th^ instar larvae and that in the controls (*P*>0.05).

#### Phenotype analysis after RNAi or DA treatment

**Figure 6:**
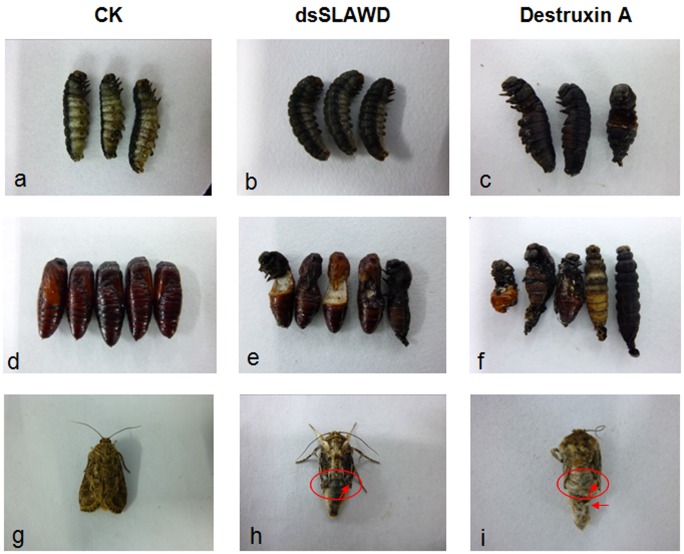
The phenotypic changes in *S. litura* after treatment with dsSLAWD or DA. (a,b,c) Prepupal phenotypic changes of *S. litura*; (d,e,f ) pupal phenotypic changes of *S. litura*; (g,h,i) adult phenotypic changes of *S. litura*. The arrows indicate shortened or deformed wing growth in the eclosion adult of *S. litura*.

**Figure 7:**
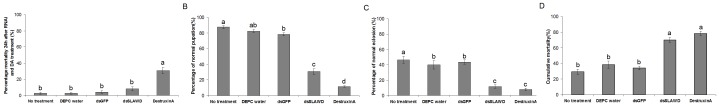
Phenotypic analysis of *S. litura* after RNAi and DA treatment. A: Percentage mortality 24 h after RNAi or DA treatment; B: Percentage of normal pupation; C: Percentage of normal eclosion; D: Cumulative mortality of *S. litura*. The different letters above the columns indicate significant differences in *SLAWD* expression after RNAi or DA treatment (*P*<0.05).

The phenotypes of *S. litura* following RNAi or DA treatment are shown in Fig. 6. *S. litura* entered the prepupal stage during the 24 h following microinjection of dsRNA or DA (Fig. 6 a,b,c). The growth and development of the insects in the three controls was normal and no unusual phenotypes were observed. However, in some of the 6^th^-stage larvae injected with dsSLAWD or DA, development was delayed and melanization of the lateral tracheae was observed in several of the larvae and prepupae. Further notable effects of DA, but not of dsSLAWD, were seriously melanization and carbonization during development of *S. litura*. The severity of these effects led to stiffening of the bodies of 6^th^-stage larvae, and resulted in larval death. Statistical analyses of growth and development showed a significant difference between the mortality in the DA group (30.83±3.96%) and that in the controls after 24 hrs (CK: 2.50±1.12%;CK DEPC water: 2.50±1.12%; CK dsGFP: 4.17±1.54%). There was no obvious difference between mortality in the RNAi group (8.33±2.47%) and that in the corresponding controls (Fig. 7 A).

At 48 hrs after dsSLAWD or DA microinjection, the prepupae of *S. litura* underwent a final molt and then entered the pupal stage. While the control pupae developed normally and reached full size (Fig. 6 d), the cephalothorax in pupae of the RNAi group was wizened and its formation was stunted. In addition, while the dorsal split was typically initiated and the new pupal cuticles were clearly visible on the head or abdomen of prepupae, the insects were unable to shed the old larval cuticle. Moreover, some prepupae successfully entered the pupal stage, but the formation and development of the head and thorax were arrested. In the DA group, the cephalic puparium were almost not formed. And the abnormal abdominal puparium prevented the prepupae from breaking through the old cuticle to enter the pupal stage; instead, they became entrapped in their larval cuticles, resulting in developmental arrest and subsequent death (Fig. 6 e,f). Statistical analyses showed that the normal pupation rates of the three controls (no treatment, DEPC water, dsGFP) were normal and similar (87.50±1.71%, 82.50±1.71%, 78.33±2.11% respectively), but the rates were significantly higher than those in the RNAi and DA groups. Furthermore, the pupation rate of the DA group was much lower than that of the RNAi group (11.67±1.05% vs. 30.83±3.52%; Fig. 7 B). Thus, the effect of mechanical injury following larval injection can be ruled out as a source of the differences between to the controls.

When dsSLAWD and DA microinjections were performed up to the eclosion stage, the insects molted normally up to the pupal stage but subsequently failed to complete the adult molt and died as pharate adults entrapped in the pupal exuvium. Other changes in the eclosion adult involved wing growth, which resulted in short or deformed wings (Fig. 6 g,h,i). The normal rates of *S. litura* eclosion by RNAi or DA treatments (11.90±2.14% and 7.93±1.67%, respectively) were significantly lower than that obtained by the control treatments (Fig. 7 C). Cumulative mortality after RNAi or DA treatments were 70.00±3.16% and 78.33±2.79%, respectively, which were significantly higher than those of the three controls (no treatment: 29.17±3.27%, DEPC water: 38.33±4.77%, dsGFP: 34.17±2.39%) (Fig. 7D).

## Discussion

### Characterization of the SLAWD Gene

The AWD gene has been identified and studied in several insect species, including *Drosophila melanogaster*, *Bombyx mori*, *Antheraea pernyi, Choristoneura parallela,* and *Diaphorina citri* [8], [17], [32]. It was initially chosen as the target gene in a study of the wing disc of *Drosophila* subjected to hybrid dysgenesis [1]. *Drosophila AWD*, a homolog of the human gene *Nm23*, regulates epithelial integrity during oogenesis, plays a role in neurotransmission and in the regulation of FGF receptor levels, and functions synergistically with *shi/dynamin* during tracheal development [9], [10], [12]. The gene also regulates of cell differentiation and motility by controlling growth factor receptor signaling, e.g., as a mediator of endocytosis [10]. In previous studies, we found that AWD amino acid sequences were also present in *S. litura* exposed to DA [31]. In this study, we cloned *SLAWD* and showed its nucleotide sequence, which is similar to that of *B. mori AWD*, encodes a protein of 178 amino acids with a calculated molecular mass of 20.10 kDa (GenBank: ABF51506.1).

The *AWD* gene is most prominently expressed in the imaginal discs and the brain after the end of larval development [33]. In this study, the pattern of *SLAWD* expression during development showed that *SLAWD* has distinct roles during the pupal and adult stages. Consistent with the expression patterns of *B. mori* and *A. pernyi AWD* genes, *SLAWD* mRNA levels were low during the larval stage [32], [34]. However, wing imaginal disc development differs in Diptera and Lepidoptera. In *Drosophila*, the determination of imaginal disc cells is initiated during embryogenesis, whereas terminal differentiation does not begin until the pupal stage [35]. In Lepidoptera, imaginal wing discs are well developed at the larval stage and do not undergo major cell rearrangement or differentiation in the early stages of larval development. This may be related to the greater requirement for AWD protein synthesis during larva–pupa metamorphosis.

AWD protein adopts oligomeric forms in its native state in other arthropods and this is likely to be the case in *S. litura* as well. By sequencing *SLAWD*, we obtained sufficient information to allow its functional analysis.

### The Roles of SLAWD in *S. litura* Growth and Wing Morphogenesis

To further elucidate the function of *SLAWD*, knockdown of its expression was achieved by injecting a specific dsRNA into *S. litura* in the 6^th^ larval stage, which is a stage that coincides with the period during which *SLAWD* transcript levels start to increase. The mutant AWD phenotype includes abnormal morphology of the wing discs and larval brain; aberrant differentiation of the wing, leg, and eye-antenna; heterogeneity in the pattern of abnormal morphology; and cell death [11]. In this study, although the specific phenotypes of the controls were not significantly different from those of the dsRNA group at the prepupal stage (Fig. 6 a,b), interestingly, the expression of *SLAWD* after RNAi treatment decreased dramatically and the normal development of *S. litura* was destroyed during the transition from the pupal prophase to the adult stage (Fig. 5). Consistent with previous studies, the main phenotypes observed following RNAi treatment were a wizened pupal cephalothorax, stunted formation and development of the cephalothorax, and shortened or deformed wing growth of the eclosion adult.

The covering of wings with scales is a unique feature of Lepidoptera and it distinguishes it from other insect species. Many factors may control wing development, such that abnormalities in *AWD* and in hormone-regulated growth and development may affect the expression levels of other genes involved in wing formation and alter the wing phenotype, respectively. Earlier studies established that AWD participates in synaptic vesicle internalization by supplying GTP required for Shi/dynamin-mediated endocytosis [36]. A role for AWD in ovarian development was demonstrated in a more complicated organ transplant experiment involving mutant imaginal discs, including wing, leg, and eye-antenna [37]. The wing defects caused by the *awd^b3^* mutation are cell autonomous [8]. According to the phenotypic analysis in the present study, silencing of *SLAWD* suppresses the development of *S. litura* pupae and causes vestigial wings in the adult (Fig. 6). These observations suggest that *SLAWD* participates in important physiological processes of wing formation and development during the pupal and eclosion stages of *S. litura*. The genes involved in wing biosynthesis are of interest as potential targets for insect pest control procedures that employ gene silencing. Although our results suggest that *SLAWD* is necessary for *S. litura* pupal and adult development, the nature of the signaling mechanism in which it is involved remains to be clarified.

### The Toxic Effect of DA on *SLAWD*

Using qRT-PCR, we found that microinjection of either RNAi or DA induced a down-regulation of *SLAWD* expression during the pupal prophase to adult stage of *S. litura*. And the phenotypes associated with DA exposure showed that it can also inhibit the pupal and wing development in *S. litura*. However, a distinction was noted between some of the phenotypes and cumulative mortality obtained in response to RNAi vs. DA treatment (Fig. 6, Fig. 7). Although DA had the toxic effect on *SLAWD* expression and the development of *S. litura*, how the insecticide alters gene expression and wing development in *S. litura* is not yet clear. The chemically induction of late larval/early pupal lethal mutations in *Drosophila* have led to the identification of many genes that are essential for normal imaginal disc development [8]. Functional proteins sensitive to DA were identified in our previous study of SL-1 cells [31]. In addition, published data from various microarray analyses suggest that wing imaginal disc development is dependent on hormones or other factors in the hemolymph [15], [16], while spatial regulation of cell adhesion in the *Drosophila* wing is mediated by β PS integrin expression [38]. In *A. pernyi*, *AWD* expression may contribute to its tolerance to high temperature [32]. Finally, wing biosynthesis in *S. litura* may be also influenced by other factors, such as the products of other genes, peritrophic-matrix-associated chitin, other juvenile hormones, and ecdysteroid, as is the case in other insects [39], [40], [41]. Thus, the specificity and effectiveness of using DA to target *SLAWD* expression will be the focus of further research.

In conclusion, this study presented a preliminary molecular characterization of the *AWD* gene in *S. litura* and an analysis of its expression patterns and function. These results further our understanding of insect *AWD* genes and provide information about the primary toxicological mechanisms of DA in *S. litura*, and ultimately, the molecular mechanisms responsible for pest outbreaks.

## Materials and Methods

### Insect Rearing

Larvae of *S. litura* were reared on a wheat germ-based artificial diet for several generations [42] at 26±1°C with a L14:D10 photoperiod in a climatic chamber with 60–70% relative humidity. The studies of the developmental expression pattern and RNAi experiments were performed with 6^th^-stage larvae of *S. litura*, whose sex was discriminated during the pupal and adult stages.

### Destruxin A

Destruxin A (DA) was separated and purified from *Metarhizium anisopliae* in our laboratory [30]. The purity was 91.5%. It was diluted with DEPC water prior to its use in the experiments.

### RNA Isolation, cDNA Synthesis, and PCR

Total RNA was extracted from *S. litura* using Trizol reagent according to the manufacturer’s specifications (Omega, Trizol RNA Kit). First-strand cDNA synthesis was carried out according to the reverse transcriptase (M-MLVRT) (TaKaRa, Japan) protocol and with oligo dT18. The primers SLAWD-F, SLAWD-R1, and SLAWD-R2 (Table 1) were designed from the amino acid sequences of SLAWD determined by MALDI-TOF-TOF MS analysis [31]. The first-strand cDNA was used as the PCR template. The PCR mix consisted of PCR buffer containing 0.1 mM dNTPs, 5 mM of each primer, and 1.0 U of HiFi-Taq DNA polymerase (Transgene, China) in a total volume of 25 µL. The first PCR amplification was performed using primers SLAWD-F and SLAWD-R1 under the following conditions: 5 min at 94°C, 30 cycles of 30 s at 94°C, 30 s at 50°C and 40 s at 72°C, and then 7 min at 72°C. The second PCR was performed using the nested primers SLAWD-F and SLAWD-R2 under the same conditions as the first PCR. Separation of the PCR products by electrophoresis revealed a weak DNA band corresponding to the expected size of approximately 300–400 bp. This band was excised from the agarose gel, purified using a DNA gel extraction kit (Omega, USA), cloned into the pMD18-T vector (Takara, Japan), and sequenced completely in both directions (Invitrogen, Guangzhou, China).

**Table 1:** PCR primers used in this study.

Primers	Primer sequence
**Degenerate primers**	
SLAWD-F	5′-AAGCCBGATGGYGTHCAACGHGG-3′
SLAWD-R1	5′-ATTTCYTTNTTDGCWGATTCHAC-3′
SLAWD-R2	5′-CCRTGVATDATGTTNCGBCCAACTT-3′
**For cDNA cloning**	
3′ *S. litura* AWD -F1	5′-GTCGGCCTCAAATTCATGTGGCCCTC-3′
3′ *S. litura* AWD -F2	5′-CGTGGCGATCTCTGCATCGAAGTTGG-3′
5′ *S. litura* AWD -R1	5′-GAGGGCCACATGAATTTGAGGCCGAC-3′
5′ *S. litura* AWD -R2	5′-CCGTTCGATAATGGTGCCAACAAGTCC-3′
**For real-time PCR**	
SLAWD-RTF	5′-CTGACTCACAACCCGGCACTATTC-3′
SLAWD-RTR	5′-CATCCGACGACTTCTTTATCTCCA-3′
Actin-RTF	5′-TCCTGGACTCCGGTGATGGTGT-3′
Actin-RTR	5′-CAGCGGTGGTGGTGAAAGAGTAAC-3′
**For dsRNA synthesis**	
dsSLAWD-F	5′- ATGATTGCCACTTTGCTATACC -3′
dsSLAWD-R	5′-TTATTCATAAACCCAGTTTTCGGC -3′
dsGFP-F	5′-AAGGGCGAGGAGCTGTTCACCG-3′
dsGFP-R	5′-CAGCAGGACCATGTGATCGCGC-3′

F: forward, R: reverse.

### Rapid Amplification of the Full-length cDNA Ends (RACE)

A full-length SLAWD cDNA was synthesized according to the manufacturer’s instructions (SMART kit, Clontech, USA). The gene-specific primers (Table 1) 5′ SLAWD -R1 and 5′ SLAWD -R2 for 5′-RACE, and 3′ SLAWD -F1 and 3′ SLAWD -F2 for 3′-RACE, were synthesized based on the cDNA sequence obtained from the identified fragment. The amplification conditions for 5′-RACE were: 6 min at 94°C, followed by 30 cycles of 30 s at 94°C, 30 s at 65°C and 40 s at 72°C, and a final extension for 10 min at 72°C. The conditions for 3′-RACE were the same. After PCR, the products were separated by electrophoresis and the DNA bands corresponding to approximately 550 bp (for 5′-RACE) and 200 bp (for 3′-RACE) were excised from the agarose gel, and purified and sequenced as described above. To confirm the validity of the obtained sequence data, each fragment was sequenced at least three times. The overlapping sequences from PCR were assembled to obtain the full-length SLAWD cDNA sequence.

### Phylogenetic Analysis of AWD Genes and Proteins

The sequence of the SLAWD cDNA was compared with that of other AWD sequences deposited in GenBank using the “BLAST” tools available on the National Center for Biotechnology Information (NCBI) website. The multiple sequence alignments of deduced amino acid sequences were performed using ClustalW [43] multiple-alignment software (http://www.ebi.ac.uk/clustalw/index.html). The phylogenetic tree was constructed using the MEGA 4 software based on the amino acid sequences of the known AWD genes [44]. A bootstrap analysis was carried out with the neighbor-joining (NJ) method [45], and the robustness of each cluster was verified in 1000 replicates.

### Developmental Expression Pattern of SLAWD

Total RNA was extracted from *S. litura* at the following developmental stages: 1-day-old eggs; 1^st^, 2^nd^, 3^rd^, 4^th^, 5^th^, and 6^th^ instar larvae; prepupae; male and female pupae; and male and female adults. The cDNA mix was diluted 1∶5 (v/v) for subsequent SYBR Green qRT-PCR amplification. qRT-PCR was performed in an CFX96™ Real-Time system (Bio-Rad, USA) using gene-specific primers (SLAWD-RTF and SLAWD-RTR; Table 1) and Platinum SYBR Green SuperMix detection (Invitrogen). As an internal control, a partial fragment of the *S. litura* actin gene was amplified from the cDNAs with the specific primers actin-RTF and actin-RTR (Table 1). qRT-PCR was carried out at 95°C for 3 min, followed by 39 cycles for SLAWD and actin of 95°C for 10 s, 55°C for 30 s, and 72°C for 30 s, plus a final extension step at 72°C for 10 min. Each sample consisted of three replicates in each plate. Dissociation curve analysis of the amplification products was performed at the end of each PCR. The PCR products were separated in a TBE-1.0% (w/v) agarose gel, stained with ethidium bromide, and visualized by UV-transillumination.

### RNA Interference (RNAi) and Destruxin A Treatments of *S. litura*

Large quantities of double-stranded RNA (dsRNA) were prepared by using T7 RNA polymerase (T7 RiboMAX™ Express RNAi system kit (Promega, USA)). The DNA fragment (537 bp) was PCR-amplified from the recombinant plasmid containing the *SLAWD* gene according to the manufacturer’s protocol using the gene-specific primers dsSLAWD-F and dsSLAWD-R with additional T7 promoter sequences for the dsRNA of *SLAWD* (Table 1). A DNA fragment (700 bp) corresponding to the green fluorescent protein (GFP) sequence was amplified as a negative control. The PCR products were purified (Omega, USA) and used to construct the dsRNA. Construction was monitored by determining the product sizes of the reactants using TBE-1.0% (w/v) agarose gel electrophoresis. The synthesized dsRNAs were dissolved in nuclease-free water and quantified by spectrophotometry.

The 39–576 bp region of SLAWD cDNA was chosen as the target region for RNAi and for DA treatment. Over the 3 days corresponding to the 6^th^ instar stage, the larvae were collected for use in the injection experiment. Five micrograms of either dsRNA or DA were injected into the side of the abdomen of the larvae using a 10 µL micro-injector. Three controls were performed: a positive control to determine the effectiveness of RNAi (microinjection of an equivalent volume of DEPC water), a negative control for monitoring the non-specific effects of RNAi (microinjection of an equivalent volume of dsGFP), and a negative control for monitoring non-microinjection effects of RNAi (no treatment). In the target gene detection experiment, each group consisted of 20 individuals with three replicates. Five insects were selected randomly from the 6^th^ instar larvae, prepupae, pupae, and adult stages after RNAi and DA treatment, and total RNA was isolated to measure transcript levels by qRT-PCR. A fragment of the *SLAWD* gene was amplified in a separate tube and served as an internal control for normalization. The PCR and thermal cycling conditions were as described above. After amplification, the qRT-PCR products were separated by TBE-1.0% (w/v) agarose gel electrophoresis and visualized as described above. In the phenotype analysis experiment, each group comprised 20 individual larvae with six replicates, which were examined daily for any visible abnormalities and mortality after injection during the 6^th^ larval, prepupal, pupal, and adult stages. Normal pupation, normal eclosion, and cumulative mortality were calculated as percentages to assess the efficiency of treatments with RNAi or DA [46], [47], [48], and the standard errors were calculated.

### Statistical Analysis

The relative expression of SLAWD was analyzed using the CFX96™ Real-Time system (Bio-Rad, USA). All the data are presented as relative mRNA expression (mean ± SEM). Data expressed as proportions, such as for malformation, survival, or cumulative mortality, were analyzed with SPSS 17.0, and significant differences was determined using Duncan’s multiple range test (DMRT).
